# Inflammatory Adipokines and Potential Oxidative Stress-Related Mechanisms Linking MASLD with Subclinical Atherosclerosis Within CKM Syndrome: A Systematic Review and Meta-Analysis

**DOI:** 10.3390/antiox15060684

**Published:** 2026-05-29

**Authors:** Cezara-Andreea Gerdanovics, Șoimița-Mihaela Suciu, Olga-Hilda Orășan, Ioana Para, Vladiana-Romina Turi, Adela-Sitar Tăut, Mircea-Vasile Milaciu, Mirela-Georgiana Perne, Teodora-Gabriela Alexescu, Lorena Ciumărnean, Alexandru Gerdanovics, Vlad-Dumitru Brata, Angela Cozma

**Affiliations:** 1 4th Department of Internal Medicine, Faculty of Medicine, “Iuliu Hațieganu” University of Medicine and Pharmacy, Republicii Street, No. 18, 400015 Cluj-Napoca, Romania; andreea.ceza.irimie@elearn.umfcluj.ro (C.-A.G.); ioana.para@umfcluj.ro (I.P.); teodora.alexescu@umfcluj.ro (T.-G.A.);; 2 Department of Physiology, “Iuliu Hațieganu” University of Medicine and Pharmacy, 1–3 Clinicilor Street, 400006 Cluj-Napoca, Romania; 3 University Clinic of Internal Medicine IV, Faculty of Medicine, “Victor Babes” University of Medicine and Pharmacy of Timisoara, 2 Eftimie Murgu Square, 300041 Timisoara, Romania; 4 2nd Department, Faculty of Nursing and Health Sciences, “Iuliu Hațieganu” University of Medicine and Pharmacy, Republicii Street, No. 18, 400015 Cluj-Napoca, Romania; 5 Department of Pathophysiology, “Iuliu Haţieganu” University of Medicine and Pharmacy, Victor Babeş Street, No. 2–4, 400012 Cluj-Napoca, Romania; 6 Clinical Rehabilitation Hospital, Viilor Street, No. 46–50, 400066 Cluj-Napoca, Romania; 7 Department of Gastroenterology, Regional Institute of Gastroenterology and Hepatology “Prof. Dr. Octavian Fodor”, 400394 Cluj-Napoca, Romania

**Keywords:** metabolic dysfunction-associated steatotic liver disease, CKM syndrome, retinol-binding protein 4, lipocalin-2, oxidative stress, subclinical atherosclerosis

## Abstract

**Background**: Metabolic dysfunction-associated steatotic liver disease (MASLD) is increasingly recognized as a systemic disorder linked to cardio-kidney–metabolic (CKM) syndrome, early vascular injury and redox imbalance. Inflammatory adipokines such as retinol-binding protein 4 (RBP4) and lipocalin-2 (LCN2) may contribute to this hepatic–vascular interplay by integrating metabolic inflammation, oxidative stress and endothelial dysfunction. Therefore, the present study aimed to investigate the contribution of the inflammatory adipokines retinol-binding protein 4 (RBP4) and lipocalin-2 (LCN2) to the hepatic–vascular interplay in MASLD within the cardio-kidney–metabolic (CKM) syndrome. **Materials and Methods**: We performed a systematic review and meta-analysis of studies evaluating circulating RBP4 and LCN2 levels in MASLD. PubMed, Scopus, and Web of Science were searched. Twenty studies were included in the qualitative synthesis, and ten in the quantitative meta-analysis. Standardized mean differences (SMDs) with 95% confidence intervals (CIs) were calculated. Vascular findings were synthesized narratively because of heterogeneity in outcomes. **Results**: Circulating RBP4 levels were significantly higher in MASLD patients than in controls (SMD = 0.64, 95% CI: 0.08 to 1.20, *p* = 0.026; I^2^ = 91.2%). LCN2 levels were also significantly elevated (SMD = 1.92, 95% CI: 0.83 to 3.00, *p* < 0.001; I^2^ = 98.0%). Compared with RBP4, LCN2 showed a larger pooled effect size, although heterogeneity remained very high. In the qualitative synthesis, adipokines, particularly LCN2, were associated with markers of vascular injury, including carotid intima–media thickness, plaque burden, arterial stiffness, endothelial dysfunction, coronary severity, and cardiovascular events. **Conclusions**: Both RBP4 and LCN2 were elevated in MASLD, supporting a link between adipokine dysregulation and hepatic metabolic dysfunction within the broader cardio-kidney–metabolic (CKM) syndrome. LCN2 appeared to better reflect the inflammatory, metabolic, and vascular burden of disease. These findings support the view of MASLD as a systemic disorder within the CKM syndrome and highlight the potential of inflammatory adipokines as non-invasive biomarkers of integrated hepatic, metabolic, and vascular dysfunction.

## 1. Introduction

Cardiovascular–kidney–metabolic (CKM) syndrome integrates dysfunctional adiposity, metabolic dysfunction, chronic kidney disease and cardiovascular disease within a multiorgan framework [1]. Poor CKM health is associated with increased morbidity, premature mortality and major cardiovascular burden, highlighting the need for improved prevention and risk stratification [2]. Within this context, metabolic dysfunction-associated steatotic liver disease (MASLD) is increasingly recognized as a systemic metabolic disorder linked to obesity, type 2 diabetes mellitus (T2DM), chronic kidney disease and cardiovascular disease through shared mechanisms including systemic inflammation, insulin resistance, dyslipidemia and endothelial dysfunction. Oxidative stress and redox imbalance also represent central mechanisms within this multiorgan interplay, contributing to hepatocellular injury, endothelial dysfunction and early atherosclerotic remodeling [2,3]. MASLD has been associated with increased risk of heart failure and CKD [4,5,6], while obesity and T2DM are major drivers of its development [7].

These same mechanisms are also central to atherosclerosis, supporting a link between MASLD and early vascular injury. Indeed, MASLD has been associated with subclinical atherosclerosis, including increased carotid intima–media thickness and arterial stiffness [8,9].

Adipokine dysregulation contributes to the chronic low-grade inflammation observed in obesity and related metabolic disorders [10,11,12]. Among the adipokines implicated in this process, retinol-binding protein 4 (RBP4) and lipocalin-2 (LCN2) have emerged as potential mediators linking metabolic dysfunction, inflammation and vascular injury. Elevated circulating RBP4 has been reported in patients with steatosis detected by imaging, although evidence in biopsy-proven disease remains limited [13]. LCN2, produced by both hepatic and immune cells, has also been proposed as a biomarker of hepatic inflammation and disease progression, although its role in steatohepatitis remains controversial [14].

However, the consistency of RBP4 and LCN2 alterations in MASLD and their relationship with subclinical atherosclerosis remain incompletely defined. Therefore, the aim of the present study was to systematically evaluate circulating levels of RBP4 and LCN2 in patients with MASLD through quantitative meta-analysis and to explore their potential role in linking hepatic metabolic dysfunction with subclinical atherosclerosis within the CKM syndrome.

## 2. Pathophysiological Background and Study Rationale

### 2.1. MASLD as a Systemic Metabolic Disease

The concept of fatty liver disease has evolved from non-alcoholic steatohepatitis (NASH), first described in 1980 [15], to non-alcoholic fatty liver disease (NAFLD) and later to metabolic dysfunction-associated fatty liver disease (MAFLD) [16,17,18]. More recently, the term metabolic dysfunction-associated steatotic liver disease (MASLD) was adopted to better reflect the central role of metabolic dysfunction in disease development [19]. MASLD is now recognized as one of the most common chronic liver diseases worldwide and includes a spectrum ranging from simple steatosis to metabolic dysfunction-associated steatohepatitis (MASH), the progressive form associated with inflammation, fibrosis and adverse clinical outcomes [16].

Beyond the liver, MASLD is increasingly regarded as a systemic metabolic disorder closely linked to cardiovascular and kidney disease. Excess and dysfunctional adipose tissue promotes inflammation, oxidative stress, insulin resistance and endothelial dysfunction, thereby contributing to both hepatic injury and early vascular damage [20,21]. In this context, redox dysregulation may act as a common biological denominator linking adipose dysfunction, hepatic steatosis, vascular injury and progression across the CKM syndrome and supports the need for an integrated, multiorgan approach to risk assessment and management [22].

### 2.2. MASLD and Early Vascular Injury

Cardiovascular disease is the leading cause of morbidity and mortality in patients with MASLD and growing evidence indicates that this association extends beyond traditional cardiometabolic risk factors [23,24]. MASLD has been linked to myocardial infarction, stroke, atrial fibrillation, heart failure with preserved ejection fraction and microvascular dysfunction, with cardiovascular risk increasing in parallel with liver disease severity [23,24]. Mechanistically, oxidative stress and redox imbalance appear to be central drivers of vascular injury in MASLD, alongside toxic lipid accumulation, systemic inflammation, endothelial dysfunction and atherogenic dyslipidemia [25,26,27,28].

Importantly, MASLD is also associated with early and subclinical vascular changes, including increased arterial stiffness and impaired coronary microvascular function [29,30]. These findings support the need for integrated cardiovascular assessment in patients with MASLD and reinforce its relevance within the CKM syndrome.

### 2.3. Inflammatory Adipokines as Pathophysiological Mediators

In MASLD, dysfunctional adipose tissue promotes a pro-inflammatory adipokine profile that contributes to insulin resistance, endothelial dysfunction and systemic metabolic imbalance [10,11,12]. Among these mediators, RBP4 and LCN2 have emerged as potential links between hepatic metabolic dysfunction and vascular injury.

RBP4 has been associated with incident MASLD, reduced disease regression, insulin resistance and atherogenic metabolic alterations, supporting its role as a mediator of both hepatic steatosis and vascular risk [31,32,33,34]. LCN2, in turn, is elevated in MASLD and MASH and has been linked to inflammatory activity, fibrosis and cardiovascular events [35,36,37].

Experimental data suggest that LCN2 may promote steatohepatitis and fibrogenesis, although its effects appear to be context-dependent [35,38]. Together, these findings support the relevance of RBP4 and LCN2 as mechanistic mediators linking MASLD with inflammation, metabolic dysfunction and early atherosclerotic injury [39].

### 2.4. Knowledge Gap in Adipokine-Related Hepatic–Vascular Interactions and Redox Dysfunction in MASLD

Despite growing interest in inflammatory adipokines in metabolic and cardiovascular disease, the available evidence in MASLD remains fragmented and inconsistent. Studies assessing circulating RBP4 and LCN2 have reported heterogeneous results, likely due to differences in study design, population characteristics, diagnostic criteria and biomarker assessment methods.

In addition, although both adipokines have been linked to metabolic dysfunction and vascular injury, their relationship with subclinical atherosclerosis in MASLD remains insufficiently defined, partly because vascular outcomes such as carotid intima–media thickness, arterial stiffness and plaque burden have been assessed inconsistently across studies. Therefore, a comprehensive synthesis of the available evidence is needed to clarify the role of RBP4 and LCN2 in MASLD, their relationship with redox-related vascular injury, and their potential contribution to early atherosclerotic changes within the CKM syndrome.

### 2.5. Aim

The aim of this study was to systematically review and quantitatively synthesize the available evidence on circulating RBP4 and LCN2 levels in patients with MASLD, with particular emphasis on their potential role as inflammatory and redox-related mediators of hepatic–vascular interplay and subclinical atherosclerosis within CKM syndrome.

## 3. Materials and Methods

### 3.1. Protocol and Reporting Guideline

This systematic review and meta-analysis was conducted for the interval from 1 January 2026 to 1 April 2026 in accordance with the Preferred Reporting Items for Systematic Reviews and Meta-Analyses (PRISMA) 2020 guidelines. The study protocol was developed in March–April 2026, in advance of data extraction and statistical analysis, and predefined the research question, eligibility criteria, and methodological approach.

Rigorous research of the papers was performed. Two main investigators performed independent literature research to identify the previously published papers. All useful papers were read by both investigators, even those with negative results.

### 3.2. Eligibility Criteria

Studies were considered eligible if they met the following inclusion criteria: (1) conducted in human subjects; (2) included adult participants (≥18 years); (3) evaluated patients with MASLD, diagnosed by imaging or histology or according to study-defined diagnostic criteria used in the original reports; (4) reported circulating levels RBP4 and/or LCN2; and (5) assessed markers of subclinical atherosclerosis, such as carotid intima–media thickness (cIMT), arterial stiffness or plaque burden.

Exclusion criteria included: (1) studies conducted in pediatric populations; (2) animal or in vitro studies; (3) case reports, reviews, editorials or conference abstracts without full data; (4) studies lacking sufficient quantitative data for analysis; and (5) systematic reviews, meta-analyses, and editorials.

### 3.3. Information Sources

A comprehensive literature search was performed using the following electronic databases: PubMed, Scopus, and Web of Science. The search aimed to identify all relevant studies published up to the date of the final search.

### 3.4. Search Strategy

The search strategy combined Medical Subject Headings (MeSH) terms and free-text keywords related to MASLD, adipokines, and subclinical atherosclerosis. The following terms were used in various combinations: “MASLD”, “MAFLD”, “NAFLD”, “non-alcoholic fatty liver disease”, “retinol-binding protein 4”, “RBP4”, “lipocalin-2”, “LCN2”, “adipokines”, “carotid intima–media thickness”, “cIMT”, “pulse wave velocity”, “arterial stiffness”, and “atherosclerosis”. Boolean operators (AND, OR) were applied to refine the search. The full search strategy is provided in Supplementary Materials [40].

### 3.5. Study Selection

**Figure 1:**
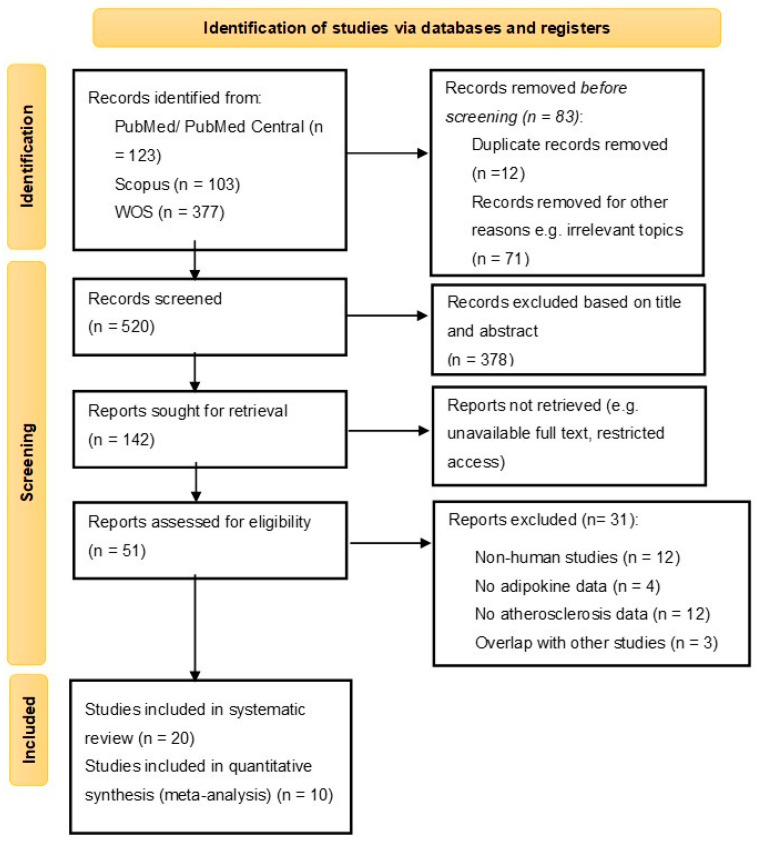
PRISMA 2020 flow diagram of study selection—Flowchart illustrating the identification, screening, eligibility assessment, and final inclusion of studies in the qualitative synthesis and quantitative meta-analysis.

All retrieved records were imported into a reference management system and duplicate entries were removed. Two independent reviewers screened titles and abstracts to identify potentially eligible studies. Full-text articles were subsequently assessed for inclusion based on predefined criteria. Disagreements between reviewers were resolved through discussion and consensus. The study selection process is illustrated using a PRISMA flow diagram (Figure 1).

### 3.6. Data Extraction

Data extraction was performed independently by two reviewers using a standardized form. The following data were collected from each study: author, title, study identification item, year, country, study design, sample size (MASLD vs. without MASLD), MASLD diagnostic method, adipokine assessed (RBP4 and/or LCN2), and assay method. For quantitative synthesis, mean values and standard deviations of circulating adipokine levels in MASLD and control groups were extracted when available; alternative summary formats were recorded and handled accordingly. Data on subclinical atherosclerosis markers, including carotid intima–media thickness, arterial stiffness, plaque burden, and vascular functional parameters, were extracted for narrative synthesis. Relevant clinical and metabolic characteristics were also recorded to support CKM-related interpretation.

### 3.7. Quality Assessment

Methodological quality was assessed independently by two reviewers using the Newcastle–Ottawa Scale (NOS) for observational studies. Discrepancies were resolved by discussion, and studies were classified as low, moderate or high quality according to NOS scores. The results are presented in a dedicated table.

### 3.8. Statistical Analysis

All analyses were performed in R software, version 4.5.3 (R Foundation for Statistical Computing, Vienna, Austria).

Standardized mean differences (SMDs) with 95% confidence intervals (CIs) were calculated for circulating RBP4 and LCN2 levels in MASLD versus control groups. Publication bias was explored by funnel plots.

Heterogeneity was assessed with Cochran’s Q test and the I^2^ statistic, and sensitivity analyses were conducted using leave-one-out methods. An I^2^ value > 25% was considered indicative of heterogeneity. Separate meta-analyses were performed for each adipokine using random-effects models. Because of heterogeneity in vascular outcomes and reporting, studies on subclinical atherosclerosis were synthesized narratively.

## 4. Results

### 4.1. Study Selection

The initial database search identified a total of 603 records from PubMed/PubMed Central (*n* = 123), Scopus (*n* = 103), and Web of Science (*n* = 377). After removal of duplicate records and those excluded for other reasons (*n* = 83), 520 records remained for title and abstract screening.

Following screening, 378 records were excluded based on title and abstract, leaving 142 reports sought for retrieval. Of these, 51 full-text articles were assessed for eligibility. A total of 31 studies were excluded due to predefined criteria, including non-human studies (*n* = 12), absence of adipokine data (*n* = 4), lack of atherosclerosis-related outcomes (*n* = 12), and overlap with other studies (*n* = 3).

Ultimately, 20 studies were included in the systematic review. Among these, 10 studies were eligible for quantitative synthesis (meta-analysis). The included studies predominantly involved populations with MASLD and varying degrees of metabolic dysfunction consistent with components of the CKM syndrome, while studies without relevant metabolic or vascular characteristics were excluded during the selection process. The study selection process is illustrated in the PRISMA flow diagram (Figure 1).

### 4.2. Study Characteristics

A total of 20 studies were included in qualitative synthesis and 10 in the quantitative meta-analysis. Most studies were observational and cross-sectional. MASLD was defined mainly by ultrasonography, while only a minority used biopsy-based diagnosis with partial stratification into simple steatosis and MASH. Differences in diagnostic confirmation across studies may have contributed to the observed heterogeneity. Also, most included studies were conducted before the adoption of the current MASLD nomenclature and therefore used earlier NAFLD or MAFLD-based diagnostic criteria. Study populations were metabolically heterogeneous, including obesity, insulin resistance, dyslipidemia and type 2 diabetes mellitus. Six studies contributed to the RBP4 meta-analysis, with ELISA being the most common assay method (Table 1).

**Table 1:** Characteristics of studies included in the RBP4 meta-analysis.

Study	Year	Country	Design	MASLD Diagnosis	Liver Phenotype	Assay	MASLD	Control	RBP4 MASLD (Mean ± SD)	RBP4 Control (Mean ± SD)	Units
Cengiz [41]	2010	Turkey	Cross-sectional	US ± biopsy	MASLD (unspecified)	ELISA	76	24	18.1 ± 0.6	18.0 ± 0.7	μg/mL
Ikizek [42]	2020	Turkey	Cross-sectional	US ± biopsy	MASLD (unspecified)	ELISA	56	25	94.6 ± 8.4	86.2 ± 7.2	μg/mL
Terra [43]	2013	Spain	Cross-sectional	Biopsy	MASLD/MASH (biopsy-proven)	Nephelometry	34	15	34.9 ± 3.8	26.5 ± 2.1	μg/mL
Polyzos [44]	2016	Greece	Cross-sectional	Biopsy (SS/MASH)	Mixed (SS + MASH)	ELISA	29	25	11.3 ± 6.5	15.9 ± 2.2	μg/mL
Chen [45]	2017	China	Cross-sectional	Ultrasound	MASLD (unspecified)	ELISA	1480	1458	37.9 ± 6.8	35.0 ± 6.7	μg/mL
Wu [46]	2008	China	Cross-sectional	Ultrasound	MASLD (unspecified)	RIA	52	50	41.3 ± 9.8	32.0 ± 8.9	μg/mL

Summary of study design, population characteristics, MASLD diagnostic method, liver phenotype, assay method, sample size, and circulating retinol-binding protein 4 levels in MASLD and control groups for studies included in the quantitative synthesis. Abbreviations: RBP4, retinol-binding protein 4; MASLD, metabolic dysfunction-associated steatotic liver disease; MASH, metabolic dysfunction-associated steatohepatitis; ELISA, enzyme-linked immunosorbent assay; RIA, radioimmunoassay; SD, standard deviation; US, ultrasound.

Because eGFR and albuminuria were inconsistently reported, and renal involvement was often not defined using standardized thresholds, formal CKM staging according to AHA criteria was not feasible. The included RBP4 populations were therefore characterized by metabolic risk profiles, with most cohorts showing features consistent with CKM stage 2 and diabetic populations suggesting more advanced phenotypes (Table 2 and Table 3).

**Table 2:** Cardiometabolic profiles of populations included in the RBP4 meta-analysis.

Study	T2DM (%)	BMI (kg/m^2^)	HOMA IR	Triglycerides (mg/dL)	HDL (mg/dL)	LDL (mg/dL)	HTN	Dyslipidemia	CKM-Related Metabolic Profile
Cengiz [41]	0%	30.1 ± 4.5	3.63 ± 3.24	187.7 ± 93.7	44.4 ± 13.4	NR	NR	Present	insulin resistance with atherogenic dyslipidemia (CKM stage 2)
Ikizek [42]	0%	25.6–27.5	3.2 ± 2.6	160 ± 104	NR	126 ± 30.8	NR	Present	insulin resistance with dyslipidemia in non-obese individuals (CKM stage 2)
Terra [43]	22.2%	49.2 ± 1.4	7.31 ± 2.39	181.9 ± 18.6	44.6 ± 2.4	NR	NR	Present	severe obesity-associated insulin resistance and dyslipidemia (CKM stage 2–3)
Polyzos [44]	NR	32.9 ± 1.6	3.45 ± 1.26	185.7 ± 34.2	49.0 ± 3.0	NR	NR	Present	obesity-related insulin resistance with dyslipidemia (CKM stage 2)
Chen [45]	8–11%	25.0 ± 3.0	NR	145 (no SD)	49 ± 11	137 ± 35	High	Present	early metabolic dysfunction with dyslipidemia and hypertension (CKM stage 2)
Wu [46]	100%	25.8 ± 2.7	7.1 ± 3.3	301 (no SD)	50 ± 15	131 ± 39	Present	Present	type 2 diabetes with severe insulin resistance and atherogenic dyslipidemia (CKM stage 3)

Overview of the cardiometabolic characteristics of study populations included in the RBP4 meta-analysis, including type 2 diabetes mellitus prevalence, body mass index, insulin resistance markers, lipid profile, hypertension, dyslipidemia, and CKM-related metabolic phenotype. Abbreviations: RBP4, retinol-binding protein 4; T2DM, type 2 diabetes mellitus; BMI, body mass index; HOMA-IR, homeostasis model assessment of insulin resistance; HDL, high-density lipoprotein; LDL, low-density lipoprotein; CKM, cardio-kidney–metabolic; HTN = Hypertension; NR, not reported.

**Table 3:** Characteristics of studies included in the LCN2 meta-analysis.

Study	Year	Country	Design	MASLD Diagnosis	Liver Phenotype	Assay	MASLD	Control	LCN2 MASLD (Mean ± SD)	LCN2 Control (Mean ± SD)	Units
Milner [47]	2009	Australia	Cross-sectional	Biopsy-proven MASLD	Mixed (SS + MASH)	ELISA	100	129	63.2 ± 26.0	48.6 ± 20.0	ng/mL
ElGhandour [14]	2024	Egypt	Case–control	Ultrasound (AASLD criteria)	MASLD (ultrasound-based)	ELISA	51	51	18.93 ± 10.03	4.66 ± 3.98	ng/mL
Ye [48]	2014	China	Cross-sectional	Ultrasound	MASLD (unspecified)	ELISA	436	467	89.67 ± 4.47	68.70 ± 3.65	ng/mL
Chawla [49]	2023	India	Case–control	Ultrasound	MASLD(graded severity)	ELISA	70	24	170.38 ± 114.43	40.46 ± 6.44	ng/mL

Summary of study design, population characteristics, MASLD diagnostic method, liver phenotype, assay method, sample size, and circulating lipocalin-2 levels in MASLD and control groups for studies included in the quantitative synthesis. Abbreviations: LCN2, lipocalin-2; MASLD, metabolic dysfunction-associated steatotic liver disease; MASH, metabolic dysfunction-associated steatohepatitis; ELISA, enzyme-linked immunosorbent assay; SD, standard deviation; AASLD, American Association for the Study of Liver Diseases.

Four studies were included in the LCN2 meta-analysis. MASLD was diagnosed mainly by ultrasonography, all studies used ELISA-based assays, and one study included biopsy-proven disease. Across studies, circulating LCN2 levels were generally higher in MASLD patients than in controls and, in some cases, were associated with disease severity or metabolic dysfunction (Table 4).

**Table 4:** Cardiometabolic profiles of populations included in the LCN2 meta-analysis.

Study	T2DM (%)	BMI (kg/m^2^)	HOMA IR	Triglycerides (mg/dL)	HDL (mg/dL)	LDL (mg/dL)	HTN	Dyslipidemia	CKM-Related Metabolic Profile
Milner [47]	28%	31.0 ± 5.0	6.3 ± 6.5	204 ± 133	50 ± 15	120 ± 39	present	present	Advanced metabolic dysfunction with insulin resistance (CKM stage 2–3)
ElGhandour [14]	31%	33.722 ± 5.581	NR	161.3 ± 74.4	47.1 ± 9.1	130.6 ± 38.8	25%	Present	Metabolic syndrome with early vascular risk (CKM stage 2)
Ye [48]	21%	26.6 ± 3.0	NR	204 ± 151	46 ± 12	124 ± 31	Elevated	Present	Early metabolic dysfunction (CKM stage 1–2)
Chawla [49]	NR	No significant difference between groups	NR	NR	NR	NR	NR	Yes	Established metabolic syndrome with steatosis severity link (CKM stage 2–3)

Overview of the cardiometabolic characteristics of study populations included in the LCN2 meta-analysis, including type 2 diabetes mellitus prevalence, body mass index, insulin resistance markers, lipid profile, hypertension, dyslipidemia, and CKM-related metabolic phenotype. Abbreviations: LCN2, lipocalin-2; T2DM, type 2 diabetes mellitus; BMI, body mass index; HOMA-IR, homeostasis model assessment of insulin resistance; HDL, high-density lipoprotein; LDL, low-density lipoprotein; CKM, cardio-kidney–metabolic; HTN = Hypertension; NR, not reported.

### 4.3. Circulating RBP4 Levels in MASLD

**Figure 2:**
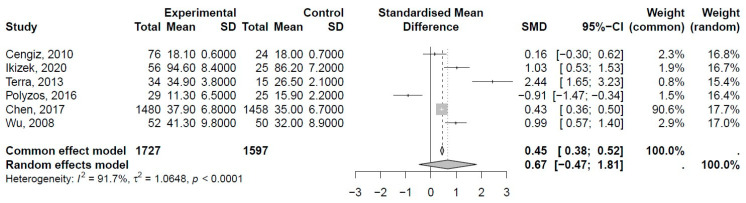
Forest plot of circulating RBP4 levels in MASLD versus controls [41,42,43,44,45,46]. Random-effects meta-analysis of the standardized mean difference in circulating retinol-binding protein 4 (RBP4) levels between patients with metabolic dysfunction-associated steatotic liver disease (MASLD) and controls. Squares represent individual study effect sizes weighted by study precision, and the diamond represents the pooled effect estimate. The pooled effect size was moderate. Abbreviations: RBP4, retinol-binding protein 4; MASLD, metabolic dysfunction-associated steatotic liver disease; SMD, standardized mean difference; CI, confidence interval. Squares represent individual study effect estimates, the horizontal lines represent the 95% confidence intervals and the diamond represents the pooled effect estimate.

Six studies were included in the RBP4 meta-analysis. Circulating RBP4 levels were significantly higher in MASLD patients than in controls (SMD = 0.64, 95% CI: 0.08 to 1.20, *p* = 0.026), although substantial heterogeneity was observed (I^2^ = 91.2%, *p* < 0.001). This substantial heterogeneity likely reflects differences in disease severity, diagnostic methods, assay methodology, and underlying metabolic profiles across the included cohorts. Because of the limited number of studies, formal subgroup analyses were not feasible. A sensitivity analysis excluding the Polyzos (2016) [44] study, which showed an opposite direction of effect, showed a pooled effect estimate of SMD = 0.96 (95% CI: 0.24 to 1.67), while the overall direction of the association remained unchanged and heterogeneity remained high (I^2^ = 89.4%). Overall, the findings suggest that RBP4 is associated with MASLD, but with marked context-dependent variability. The forest plot is shown in Figure 2.

### 4.4. Circulating Lipocalin-2 Levels in MASLD

**Figure 3:**
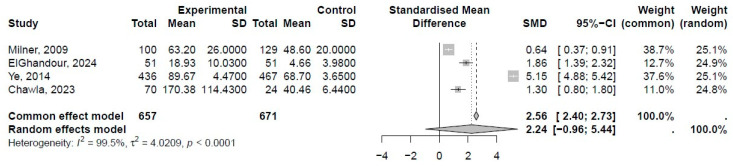
Forest plot of circulating LCN2 levels in MASLD versus controls [14,47,48,49]. Squares indicate individual study effect estimates, horizontal lines indicate 95% confidence intervals, and the diamond indicates the pooled effect estimate. SMD, standardized mean difference; CI, confidence interval; LCN2, lipocalin-2; MASLD, metabolic dysfunction-associated steatotic liver disease.

Four studies were included in the LCN2 meta-analysis. Circulating LCN2 levels were significantly higher in MASLD patients than in controls (SMD = 1.92, 95% CI: 0.83 to 3.00, *p* < 0.001), with considerable heterogeneity across studies (I^2^ = 98.0%, *p* < 0.001). The considerable heterogeneity observed in the LCN2 meta-analysis likely reflects differences in study populations, disease severity and metabolic burden. Because of the limited number of studies and the uneven reporting of key study characteristics, formal subgroup analyses were not feasible. Across the included studies, circulating LCN2 levels were consistently higher in MASLD patients than in controls, although the magnitude of this increase varied across populations. Several studies also suggested associations with steatosis severity, metabolic syndrome features, or broader metabolic dysfunction. Taken together, these findings support LCN2 as a marker of inflammatory and metabolic burden in MASLD, although heterogeneity remained very high (Figure 3).

Across the included studies, circulating LCN2 levels were consistently higher in MASLD patients than in controls, although the magnitude of this increase varied across populations. Several studies also suggested associations with steatosis severity, metabolic syndrome features, or broader metabolic dysfunction. Taken together, these findings support LCN2 as a marker of inflammatory and metabolic burden in MASLD, although heterogeneity remained very high. According to Cohen’s conventional thresholds for standardized mean differences, the pooled effect size for RBP4 (SMD = 0.64) may be interpreted as moderate, whereas the pooled effect size for LCN2 (SMD = 1.92) indicates a large difference between MASLD patients and controls.

### 4.5. Adipokines and Subclinical Atherosclerosis

The association between adipokines and subclinical atherosclerosis was evaluated in a subset of studies and is summarized in Table 5. Because vascular endpoints and reporting methods were highly heterogeneous, quantitative synthesis was not feasible. Overall, both RBP4 and LCN2 were associated with vascular abnormalities, although the evidence was more extensive for LCN2, which was linked to carotid plaque, arterial stiffness, coronary severity, and cardiovascular events. By contrast, evidence for RBP4 was more limited and heterogeneous. These findings suggest that adipokines, particularly LCN2, may reflect vascular injury in metabolically unfavorable populations.

Elevated LCN2 levels were linked to carotid plaque, arterial stiffness, coronary severity, and cardiovascular events across several metabolically unfavorable populations. In contrast, findings for RBP4 were more limited and heterogeneous, with positive associations reported mainly for carotid intima–media thickness. Although these findings are relevant within the CKM framework, most vascular studies did not explicitly confirm MASLD. Therefore, the hepatic–vascular interpretation should be considered supportive rather than definitive. Nevertheless, the available data suggest that adipokines, particularly LCN2, may be associated with subclinical and clinical atherosclerosis in metabolically high-risk populations sharing key features with MASLD. Taken together, these data suggest that adipokines, particularly LCN2, may be associated with subclinical and clinical atherosclerosis in populations with adverse cardiometabolic profiles.

### 4.6. Risk of Bias Assessment of Included Studies

The methodological quality of the included studies was assessed using the Newcastle–Ottawa Scale (NOS). Overall, most studies were of moderate quality, with limitations primarily related to sample size, cross-sectional design, and potential selection bias (Table 6).

**Table 6:** Risk of bias assessment of included studies using the Newcastle–Ottawa Scale (NOS).

Study	Year	Design	Selection (0–4)	Comparability (0–2)	Outcome (0–3)	Total (0–9)	Quality
Cengiz [41]	2010	Cross-sectional	3	1	2	6	Moderate
Ikizek [42]	2020	Cross-sectional	3	1	2	6	Moderate
Terra [43]	2013	Cross-sectional	4	1	2	7	High
Polyzos [44]	2016	Cross-sectional	4	2	2	8	High
Chen [45]	2017	Cross-sectional	3	1	2	6	Moderate
Wu [46]	2008	Cross-sectional	3	1	2	6	Moderate
Milner [47]	2009	Cross-sectional	4	2	2	8	High
ElGhandour [14]	2024	Case–control	3	2	2	7	High
Ye [48]	2014	Cross-sectional	3	1	2	6	Moderate
Chawla [49]	2023	Case–control	3	2	2	7	High

Methodological quality assessment of studies included in the quantitative synthesis, with reporting of selection, comparability, outcome domains, total score, and overall quality category. Abbreviations: NOS, Newcastle–Ottawa Scale.

**Figure 4:**
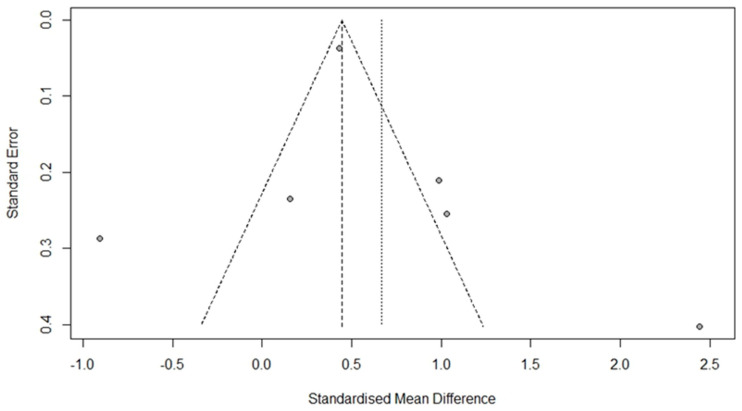
Funnel plot for the RBP4 meta-analysis. Funnel plot assessing potential small-study effects and publication bias in studies included in the meta-analysis of circulating retinol-binding protein 4 levels in patients with metabolic dysfunction-associated steatotic liver disease. The dots represent the individual studies, while the dashed lines indicate the confidence limits/pseudo-confidence region of the funnel plot.

Overall, the methodological quality of included studies ranged from moderate to high. Most studies achieved scores between 6 and 8 points on the Newcastle–Ottawa Scale, reflecting adequate selection of study populations and outcome assessment. Higher-quality studies were generally those with biopsy-confirmed MASLD or well-defined case–control designs (Figure 4).

**Figure 5:**
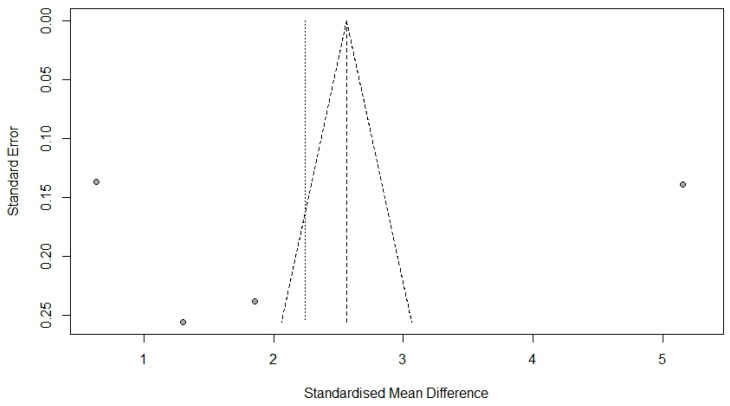
Funnel plot for the LCN2 meta-analysis. Funnel plot assessing potential small-study effects and publication bias in studies included in the meta-analysis of circulating lipocalin-2 levels in patients with metabolic dysfunction-associated steatotic liver disease. The dots represent the individual studies included in the meta-analysis, while the dashed lines indicate the pseudo-95% confidence limits of the funnel plot.

Publication bias for the LCN2 meta-analysis was assessed using a funnel plot, as shown in Figure 5.

Publication bias was explored using funnel plots. Interpretation of funnel plots is limited by the small number of included studies; therefore, the presence of publication bias cannot be excluded. Egger’s regression was not performed because of the limited number of studies.

## 5. Discussion

In this systematic review and meta-analysis, both RBP4 and LCN2 were elevated in patients with MASLD compared with controls, supporting their relevance to the metabolic and inflammatory disturbances that characterize this condition. However, the two adipokines showed distinct patterns.

RBP4 was significantly associated with MASLD, but with substantial between-study heterogeneity, suggesting that its levels may be strongly influenced by the underlying metabolic context. This interpretation is consistent with previous reports showing that the association between RBP4 and MASLD in patients with T2DM remains inconsistent [46,58], despite evidence that MASLD tends to be more severe in diabetic individuals, with higher proportions of steatohepatitis and cirrhosis [59]. Within the CKM syndrome, this is particularly relevant, as T2DM reflects a more advanced metabolic phenotype characterized by greater insulin resistance, systemic inflammation, endothelial dysfunction and cardiovascular risk. In our meta-analysis, the included RBP4 cohorts also supported this pattern, with T2DM prevalence ranging from 0% to 100% and consistently abnormal insulin resistance markers when reported, suggesting that elevated RBP4 may reflect not only hepatic steatosis itself, but also the broader burden of metabolic dysfunction. This is further supported by previous studies linking higher RBP4 levels to less favorable lipid parameters and greater adiposity [60,61,62]. Taken together, these findings suggest that RBP4 is associated with MASLD, but that it may be more closely related to the metabolic substrate of disease than to liver involvement alone. By contrast, LCN2 showed a larger pooled effect size across MASLD populations, although heterogeneity remained very high. Previous studies have linked higher LCN2 levels to obesity-related parameters such as BMI and waist circumference [63], although not all findings are fully explained by adiposity alone, as higher LCN2 levels have also been reported with increasing liver disease severity even in the absence of significant BMI differences [49]. In our review, this pattern was reinforced by studies suggesting associations between LCN2 and steatosis severity, adverse metabolic phenotypes, and biopsy-characterized inflammatory liver disease, supporting its role as a marker of both metabolic and hepatic burden.

Within the CKM syndrome, the available data also suggest a possible stage-related pattern in adipokine behavior. In earlier CKM phenotypes, characterized mainly by obesity, insulin resistance, and dyslipidemia without overt renal or cardiovascular disease, both RBP4 and LCN2 appear to reflect an adverse metabolic background, although RBP4 may be more closely linked to the underlying insulin-resistant substrate. In more advanced phenotypes, particularly those including T2DM, renal involvement, or established cardiovascular disease, LCN2 was more frequently associated with vascular abnormalities and clinical cardiovascular outcomes, suggesting that it may better capture the transition from metabolic dysfunction to overt vascular injury. Although formal AHA-based CKM staging was not feasible across all included studies because of incomplete renal data, this gradient supports the view that RBP4 may predominantly reflect earlier metabolic disturbance, whereas LCN2 may better track more advanced inflammatory and vascular involvement across the CKM syndrome.

In addition, the qualitative vascular synthesis showed that adipokines, particularly LCN2, were associated with markers of subclinical and overt vascular injury, including carotid intima–media thickness, plaque burden, arterial stiffness, endothelial dysfunction, coronary severity, and cardiovascular events. Although MASLD was not explicitly assessed in all vascular studies, most included populations shared key features of the MASLD phenotype, such as obesity, insulin resistance, T2DM, and renal or cardiovascular comorbidity, making these findings highly relevant within the CKM syndrome. Overall, our results support the view that MASLD should be regarded as part of a broader cardiometabolic disorder and suggest that LCN2 may better reflect the inflammatory and vascular dimension of disease, whereas RBP4 appears to be more closely linked to its metabolic background.

### 5.1. Pathophysiological Interpretation of Adipokine-Related Hepatic–Vascular Interplay in MASLD

The association between MASLD, inflammatory adipokines, and subclinical atherosclerosis may be understood within a shared redox–inflammatory framework. Redox dysregulation may act as a mechanistic bridge between hepatic steatosis and vascular injury, as oxidative stress contributes to hepatocellular lipid accumulation, inflammatory signaling, impaired nitric oxide bioavailability, endothelial dysfunction and early atherosclerotic remodeling.

MASLD develops on a background of insulin resistance, dysfunctional adiposity, oxidative stress, and endothelial impairment, all of which promote both hepatocellular injury and atherosclerotic remodeling. In our study, both RBP4 and LCN2 were elevated in MASLD, but LCN2 appeared to show a stronger relationship with vascular involvement, suggesting that these adipokines may reflect different aspects of the hepatic–vascular interaction within the CKM syndrome.

LCN2 may better reflect vascular inflammation than RBP4 because its role appears to extend beyond systemic metabolic dysfunction to more direct vascular and plaque-related processes. It has been linked to neutrophil recruitment, inflammatory signaling and stabilization of MMP-9 activity, mechanisms that may favor extracellular matrix degradation, fibrous cap weakening, and plaque instability [64]. This may help explain why LCN2 was more frequently associated with vascular abnormalities and cardiovascular outcomes, whereas RBP4 appeared to be more closely related to the broader metabolic substrate, particularly insulin resistance and dyslipidemia. Accordingly, RBP4 and LCN2 may be interpreted not only as inflammatory and metabolic markers, but also as indirect indicators of redox-sensitive pathways linking hepatic dysfunction with vascular damage.

The mechanisms linking RBP4 to MASLD are not fully clarified, but available data suggest effects on hepatic lipid metabolism through retinoid-sensitive transcriptional pathways involving RAR, RXR, and PPARs [65], as well as direct promotion of hepatocellular lipogenesis via SREBP-1 activation through a PGC-1β-dependent pathway [66]. LCN2, in turn, has been implicated in obesity-related metabolic complications and hepatic injury [67], although its effects appear context-dependent, with studies suggesting both promotion of steatohepatitis through CXCR2-mediated inflammatory signalling [35] and protective hepatocyte-derived actions on lipolysis, lipid peroxidation, and apoptosis [67]. Both adipokines may also contribute to vascular injury through redox-sensitive mechanisms, including increased ROS production, reduced nitric oxide bioavailability, NF-κB-mediated inflammation and matrix remodeling. For RBP4, these effects appear to be mainly indirect and pro-atherogenic [38,68,69], whereas LCN2 may exert broader inflammatory and metabolic vascular actions, including NLRP3 activation, adipose inflammation, insulin resistance, foam-cell formation, and altered HDL flux [70,71]. Overall, these data support the biological plausibility of adipokine dysregulation as a link between MASLD and subclinical atherosclerosis and suggest that inflammatory adipokines, particularly LCN2, may act as accessible surrogates of redox-active hepatic–vascular crosstalk, thereby better reflecting the inflammatory and vascular burden of disease. Although oxidative stress was not directly measured in the included studies, the available evidence supports redox dysregulation as a plausible mechanistic interface linking adipokine imbalance, MASLD progression and vascular injury.

### 5.2. Implications for Redox-Related Risk Stratification in MASLD and CKM Syndrome

From the perspective of antioxidant and redox-oriented research, biomarkers that capture both inflammatory and oxidative components of disease may be particularly valuable in identifying patients with biologically active and clinically unfavorable MASLD.

As previously noted, liver biopsy and histopathological assessment remain necessary for distinguishing between different stages of MASLD, particularly in patients with more advanced disease. This is especially important because many individuals with MASLD remain asymptomatic or undiagnosed until later stages. Although several non-invasive approaches are currently available, including laboratory testing, imaging modalities and circulating biomarkers, none can yet fully capture the entire histological spectrum of the disease. Therefore, the identification of reliable non-invasive biomarkers capable of reflecting both disease presence and severity remains an important clinical priority.

In this context, LCN2 appears to be a promising candidate biomarker for the non-invasive assessment of clinically relevant MASLD. In our meta-analysis, circulating LCN2 levels were significantly higher in patients with MASLD than in controls, with a larger pooled effect size than that observed for RBP4. Moreover, the studies included in our review suggest that LCN2 may also be linked to disease severity and to more adverse metabolic and vascular phenotypes, supporting its potential relevance as a marker of more active or clinically unfavorable disease. Since LCN2 can be measured in readily accessible biological fluids, it may represent a useful non-invasive biomarker for identifying patients with MASLD who warrant closer metabolic and cardiovascular assessment [72]. However, given the substantial heterogeneity across studies, its clinical utility should be confirmed in larger, standardized cohorts before routine implementation.

Future research should focus on prospective studies with standardized biomarker assessment and integrated liver–vascular phenotyping to determine whether RBP4 and LCN2 provide incremental value for risk stratification in MASLD.

### 5.3. Limitations

Several limitations should be acknowledged. The number of studies included in the quantitative synthesis was relatively small, particularly for LCN2, limiting statistical power, precluding meaningful subgroup analyses, and reducing the reliability of publication bias assessment. Substantial heterogeneity was also observed across studies, likely reflecting differences in study populations, sample size, geographic background, metabolic profile, proportion of patients with obesity or type 2 diabetes mellitus, liver disease severity, and assay methodology. Because of the limited number of studies and the non-uniform reporting of relevant study-level characteristics, formal subgroup analyses and meta-regression by diagnostic method, diabetes status, assay type, or geographic region were not feasible. In addition, most studies were observational and predominantly cross-sectional, preventing conclusions regarding causality or temporal relationships between adipokine alterations, MASLD progression, and vascular injury. Accordingly, the pooled estimates should be interpreted cautiously and viewed as supportive rather than definitive, particularly in light of the substantial heterogeneity across studies. In addition, most studies were observational and predominantly cross-sectional, preventing conclusions regarding causality or temporal relationships between adipokine alterations, MASLD progression, and vascular injury.

Important confounders such as medication use and alcohol-related factors were not consistently reported or adjusted for across the included studies, which may have influenced the observed associations and MASLD was not uniformly defined, as many studies predated the current nomenclature and relied mainly on ultrasonography, while only a minority included biopsy-proven disease or histological stratification. Most included studies used earlier NAFLD or MAFLD-based diagnostic criteria rather than contemporary MASLD definitions. In addition, diagnostic approaches were not fully uniform across studies. Although most studies used imaging-based definitions and a minority included biopsy-confirmed disease, some studies relied on broader study-defined diagnostic criteria, which may have contributed to heterogeneity and may limit generalizability. Because of the limited number of studies and the uneven distribution of diagnostic categories, subgroup analyses according to diagnostic method were not feasible. Accordingly, the impact of diagnostic criteria on the pooled estimates could not be formally assessed, which may limit the generalizability of the findings to populations defined according to contemporary MASLD criteria.

The included populations were also metabolically heterogeneous, ranging from early metabolic dysfunction to overt diabetes and more advanced cardiometabolic phenotypes, raising the possibility that circulating RBP4 and LCN2 levels were influenced by the broader metabolic background rather than liver disease alone. In addition, formal CKM staging according to AHA criteria was limited by the inconsistent reporting of kidney-related variables, as many studies did not provide standardized definitions or cut-offs for renal involvement such as eGFR or albuminuria. Kidney function represents an additional important confounder, particularly for LCN2, as impaired renal clearance may independently increase circulating levels. eGFR and albuminuria were inconsistently reported across the included studies, and renal status was not uniformly addressed in the eligibility criteria or adjusted for analytically. None of the included studies adjusted for kidney function, which independently influences circulating LCN2 levels and may have biased effect estimates. Therefore, elevated LCN2 may in part reflect renal impairment rather than MASLD itself, which may have biased the observed effect estimates toward an overestimation of the association. Moreover, most vascular studies did not explicitly confirm MASLD, which limits the strength of the hepatic–vascular interpretation. Future prospective studies should apply unified CKM staging with harmonized hepatic, renal and vascular phenotyping in order to better define the place of inflammatory adipokines across the MASLD–CKM spectrum.

Finally, the vascular component remained qualitative because the included studies assessed heterogeneous endpoints—including cIMT, plaque burden, pulse wave velocity, endothelial function, coronary severity, and cardiovascular events—using different methodologies, which precluded quantitative synthesis.

Future studies should include larger prospective cohort designs with more detailed assessment of medications, alcohol exposure and other relevant metabolic and vascular confounders.

## 6. Conclusions

In conclusion, the present systematic review and meta-analysis suggests that inflammatory adipokines may reflect a relevant pathophysiological link between MASLD and subclinical atherosclerosis. Both RBP4 and LCN2 were elevated in MASLD, supporting a link between adipokine dysregulation, hepatic metabolic dysfunction and redox-related vascular injury within CKM syndrome. LCN2 appeared to better reflect the inflammatory, oxidative, metabolic and vascular burden of disease. These findings highlight the potential of inflammatory adipokines as non-invasive biomarkers of integrated hepatic and vascular dysfunction in redox-driven cardiometabolic disease. Although LCN2 and RBP4 may represent promising non-invasive biomarkers, further prospective and methodologically standardized studies are required before their routine clinical application can be established.

## Figures and Tables

**Table 5:** Relationship between adipokines, MASLD and atherosclerosis in patients with CKM.

Study	Year	Population	Adipokine	Vascular Marker	Main Finding	Liver Phenotype (MASLD Spectrum)	CKM Phenotype (AHA-Based Classification)
Xiao [50]	2013	T2DM patients	RBP4, LCN2	Carotid IMT (ultrasound)	Both adipokines ↑ in patients with subclinical atherosclerosis; correlated with IMT	Metabolic population (MASLD likely, not assessed)	Advanced CKM phenotype (CKM Stage 3–4 spectrum): T2DM-associated metabolic dysfunction with subclinical atherosclerosis
Gan [51]	2022	T2DM ± nephropathy	LCN2	Carotid plaque (ultrasound)	LCN2 ↑ in patients with plaque and renal damage	Metabolic + renal population (MASLD likely, not assessed)	CKM phenotype with combined metabolic and renal involvement (CKM Stage 3–4 spectrum)
El Mulla [52]	2021	ED patients (obese/non-obese)	LCN2	CIMT (ultrasound)	LCN2 ↑ with obesity and correlates with CIMT	Obese population (MASLD likely, not assessed)	Early metabolic–vascular CKM phenotype (CKM Stage 2–3 transition)
Soylu [53]	2014	CAD patients	LCN2	Aortic stiffness (PWV)	LCN2 ↑ and correlates with stiffness index	Cardiovascular cohort (MASLD not assessed)	Cardiovascular-dominant CKM phenotype (CKM Stage 4)
Schinzari [54]	2023	Obese ± metabolic abnormalities	LCN2, RBP4	Endothelial function (vasodilation)	LCN2 inversely correlates with vasodilation	Metabolic abnormalities (MASLD likely, not assessed)	Early CKM phenotype with endothelial dysfunction (CKM Stage 2–3 transition)
Li [55]	2019	CAD patients	LCN2	Coronary severity (SYNTAX score)	LCN2 correlates with disease severity	CAD cohort (MASLD not assessed)	Established cardiovascular CKM phenotype (CKM Stage 4)
Gencer [56]	2014	PCOS patients	LCN2	CIMT (ultrasound)	CIMT ↑ but LCN2 ↓ vs. control	PCOS (metabolic phenotype; MASLD possible)	Metabolic CKM phenotype without overt vascular disease (CKM Stage 2)
Giaginis [57]	2010	Carotid atherosclerosis	LCN2	Carotid plaque (ultrasound)	LCN2 associated with risk factors (renal, HTN)	Atherosclerosis cohort (MASLD not assessed)	Intermediate CKM phenotype with subclinical atherosclerosis (CKM Stage 2–3 transition)
Wu [37]	2014	Population cohort	LCN2	Cardiovascular events (clinical outcome)	LCN2 predicts incident CVD	General population (MASLD not assessed)	Longitudinal CKM phenotype with predictive value for cardiovascular events (CKM Stage 3–4 spectrum)

Narrative summary of studies evaluating the association of retinol-binding protein 4 and lipocalin-2 with markers of subclinical or clinical vascular injury, including carotid intima–media thickness, plaque burden, arterial stiffness, endothelial dysfunction, coronary severity, and cardiovascular events, in populations relevant to the cardio-kidney–metabolic spectrum. Abbreviations: RBP4, retinol-binding protein 4; LCN2, lipocalin-2; MASLD, metabolic dysfunction-associated steatotic liver disease; CKM, cardio-kidney–metabolic; CIMT, carotid intima–media thickness; T2DM, type 2 diabetes mellitus; PWV, pulse wave velocity; CAD, coronary artery disease; PCOS, polycystic ovary syndrome; CVD, cardiovascular disease; AHA, American Heart Association. “↑” indicates an increased value/level, “↓” indicates a decreased value/level.

## Data Availability

No new data were created or analyzed in this study. Data sharing is not applicable to this article.

## Supplementary Materials

The following supporting information can be downloaded at: https://www.mdpi.com/article/10.3390/antiox15060684/s1. PRISMA 2020 Checklist [40].

## Abbreviations

The following abbreviations are used in this manuscript:

AASLD	American Association for the Study of Liver Diseases
AHA	American Heart Association
BMI	Body mass index
CAD	Coronary artery disease
CI	Confidence interval
cIMT	Carotid intima–media thickness
CKD	Chronic kidney disease
CKM	Cardio-kidney–metabolic
CVD	Cardiovascular disease
ELISA	Enzyme-linked immunosorbent assay
eGFR	Estimated glomerular filtration rate
HDL	High-density lipoprotein
HOMA-IR	Homeostasis model assessment of insulin resistance
I^2^	I-squared statistic
LCN2	Lipocalin-2
LDL	Low-density lipoprotein
MAFLD	Metabolic dysfunction-associated fatty liver disease
MASH	Metabolic dysfunction-associated steatohepatitis
MASLD	Metabolic dysfunction-associated steatotic liver disease
MeSH	Medical Subject Headings
NAFLD	Non-alcoholic fatty liver disease
NASH	Non-alcoholic steatohepatitis
NGAL	Neutrophil gelatinase-associated lipocalin
NOS	Newcastle–Ottawa Scale
NR	Not reported
PCOS	Polycystic ovary syndrome
PPAR	Peroxisome proliferator-activated receptor
PRISMA	Preferred Reporting Items for Systematic Reviews and Meta-Analyses
PWV	Pulse wave velocity
RBP4	Retinol-binding protein 4
RIA	Radioimmunoassay
ROS	Reactive oxygen species
SD	Standard deviation
SMD	Standardized mean difference
SS	Simple steatosis
SYNTAX	Synergy Between Percutaneous Coronary Intervention with Taxus and Cardiac Surgery
T2DM	Type 2 diabetes mellitus
US	Ultrasound
